# TNF-α promotes breast cancer cell migration and enhances the concentration of membrane-associated proteases in lipid rafts

**DOI:** 10.1007/s13402-016-0280-x

**Published:** 2016-04-04

**Authors:** Dominika Wolczyk, Magdalena Zaremba-Czogalla, Anita Hryniewicz-Jankowska, Renata Tabola, Krzysztof Grabowski, Aleksander F. Sikorski, Katarzyna Augoff

**Affiliations:** 1 Laboratory of Cytobiochemistry, Biotechnology Faculty, University of Wroclaw, Wroclaw, Poland; 2 Department of Gastrointestinal and General Surgery, Wroclaw Medical University, Wroclaw, Poland

**Keywords:** TNF-α, DPPIV/CD26, FAP-α/F19/seprase, MT1*-*MMP/MMP14, MMP9, MMP2, DRM

## Abstract

**Purpose:**

Tumor progression is associated with cell migration, invasion and metastasis. These processes are accompanied by the activation of specific proteases that are either linked to cellular membranes or are secreted into extracellular spaces. TNF-α is known to play an important role in various aspects of tumor progression. The aim of this work was to assess the effect of TNF-α on the migration of breast cancer cells and, in addition, to assess its association with the location of membrane-associated proteases in lipid rafts.

**Methods:**

Wound scratch healing and Transwell migration assays were used to study the effect of TNF-α on the migration of both hormone-dependent and hormone-independent breast cancer*-*derived cells, i.e., MCF7 and MDA-MB-231, respectively. The expression and secretion of three matrix metalloproteases, MMP9, MMP2 and MT1-MMP, and two dipeptidyl peptidases, CD26 and FAP-α, was investigated using RT-PCR, Western blotting and gelatin zymography. In addition, activation of the MAPK/ERK signaling pathway was investigated by Western blotting.

**Results:**

We found that a TNF-α-induced enhancement of breast cancer cell migration was accompanied by an increased secretion of MMP9, but not MMP2, into the culture media. We also found that TNF-α upregulated the expression of the dipeptidyl peptidases CD26 and FAP-α in a dose-dependent manner and, in addition, enhanced the concentration of all five proteases in lipid rafts in the breast cancer-derived cells tested, regardless of cell type. Furthermore, we found that TNF-α activated the MAPK/ERK signaling pathway by increasing the ERK1/2 phosphorylation level. Application of the MEK/ERK1/2 inhibitor U-0126 resulted in down-regulation of TNF-α-induced MMP9 secretion and abrogation of the enhanced concentration of proteases in the lipid rafts.

**Conclusions:**

From our results we conclude that TNF-α-induced activation of the MAPK/ERK signaling pathway may promote breast cancer cell migration via both upregulation of MMP9, CD26 and FAP-α and concentration of these proteases, as also MT1-MMP and MMP2, in the lipid rafts. TNF-α may serve as a potential therapeutic target in breast cancers susceptible to TNF-α stimulation.

## Introduction

Caner progression depends on various cellular events, including cytokine-dependent promotion of cell migration and the production and activation of different proteolytic enzyme systems. A major step in invasion and metastasis is the destruction of a dense network of structural proteins that act as a natural barrier. This process is associated with the activation of specific proteases that are either linked to cellular membranes or are secreted into extracellular spaces [1]. Proteases are crucial players, not only in the degradation of extracellular matrix (ECM) components, but also in the processing of diverse soluble bioactive mediators and the shedding of a large variety of cell surface proteins. The production of proteases is tightly regulated by various signals mediated by cytokines, growth factors and mechanical stress [1, 2]. Many cells increase their proteolytic activity in response to inflammatory cytokines, such as tumor necrosis factor α (TNF-α), which is frequently upregulated in human epithelial malignancies such as breast cancer [3–5]. TNF-α exerts diverse functions in the biology of cancer. In addition to causing cell death, it can stimulate cancer cell survival and proliferation as well as promote angiogenesis, tumor cell migration and invasion [6].

The role of matrix metalloproteinases (MMPs) and dipeptidyl peptidases (DPPs) in invasive and metastatic processes has amply been documented [1, 7–9]. MMPs are zinc- and calcium-dependent peptidases essential for ECM reorganization. As such, they can determine the aggressiveness/invasiveness of cancer cells. Membrane-type metalloproteinase 1 (MT1-MMP, also known as MMP14) is a member of a membrane-anchored MMP subfamily [10]. It is synthesized as a 64-kDa pro-enzyme that undergoes furin-catalyzed proteolytic cleavage resulting in an active 54-kDa protein. This protein cannot only degrade ECM fibrillar components, proteoglycans or cell surface receptors and cell adhesion molecules, but can also act as a specific initiator of zymogen activation of other MMPs, including metalloproteinase-2 (MMP2/gelatinase A) and, indirectly, metalloproteinase-9 (MMP9/gelatinase B) [2, 11, 12]. MMP2 and MMP9, two members of the gelatinase subfamily of MMPs, are thought to particularly play a role in the early steps of cancer cell invasion and tumor vascularization since they cleave type IV collagen, laminin and elastin, which are the major components of the basement membrane (BM). Similar to all other soluble MMPs, MMP2 and MMP9 are secreted as inactive pro-enzymes. Their latent forms can be activated through an autocatalytic reaction or through cleavage by other proteases, including serine proteases. In addition to their ability to directly degrade extracellular components, MMP2 and MMP9 are known to affect cell signaling by releasing the active ectodomain of the fibroblast growth factor receptor 1 (FGFR-1) or by activating transforming growth factor β1 (TGF-β1). MMP2 and MMP9 are secreted into the extracellular space, but they can also become localized at the surface of tumor cells. By doing so, they are able to associate with the MT1-MMP/TIMP-2 complex, αvβ3 and α3β1 integrins, and/or CD44 and, as such, to degrade the local ECM, which enhances the invasive capacity of cancer cells [2, 7, 8, 13].

CD26 (dipeptidyl peptidase IV, DPPIV) and fibroblast activation protein-α (FAP-α, or F19 cell surface antigen, also known as seprase) are two members of the family of DPPs, i.e., serine proteases exhibiting a post-proline dipeptidyl aminopeptidase activity. In their active constitutions, both enzymes form transmembrane homo- or hetero-dimeric glycoprotein complexes with a molecular weight of ~200 kDa. The *FAP-α* and *DPPIV* genes are located close together on chromosome 2 (q23 and q24.3, respectively). The encoded proteins share 52 % amino acid sequence identity, but they differ in their cellular and substrate specificity. CD26 is a ubiquitously expressed peptidase that releases a number of biologically active peptides involved in cellular growth, migration, invasion, neovascularization and immune system activation. FAP-α cleaves larger proteins and shows a collagen type I-specific gelatinase activity. FAP-α is selectively expressed by myofibroblast-like cells within the tumor stroma, by fibrotic and granulation tissue cells and by several types of cancer cells. MT1-MMP and both gelatinases, as well as CD26 and FAP-α, have been found to localize to sites of focal ECM degradation, i.e., in specialized F-actin-based membrane protrusions denoted as invadopodia or cell-matrix adhesive structures enriched in ordered membranous micro-domains known as lipid rafts [14–16].

Lipid rafts are more tightly packed than its surrounding non-raft lipid bilayer and they can sequester specific proteins involved in cell-cell interactions, actin cytoskeleton organization, cell-ECM adhesion and membrane dynamics. As such, they can serve as platforms for membrane trafficking, signaling and polarization. Lipid rafts organize many signaling proteins, including integrin and non-integrin receptors, and various enzymes such as kinases, phosphatases or membrane-associated proteases to regulate the motility of cells. The localization of these proteins inside or outside the lipid rafts determines their functional activities. Lipid rafts are tightly linked to the targeted delivery, organization and activation of specialized molecules implicated in cancer metastasis at the leading edge of migrating cells [16–21]. Lipid rafts have been shown to be crucial for the formation and extension of membrane protrusions, and lipid raft-disrupting reagents have been found to decrease the migratory potential of tumor cells [22, 23].

It is still unclear, however, whether a cytokine-dependent increase of cancer cell migration and invasion is related to enhanced ECM-degrading activities via the accumulation of proteolytic enzymes in lipid rafts. To address this issue we stimulated estrogen-dependent MCF7 breast cancer-derived cells and highly invasive, hormone-independent MDA-MB-231 breast cancer-derived cells with TNF-α and subsequently assessed changes in cell migration in conjunction with the levels of two dipeptidyl peptidases, FAP-α and CD26, and three metalloproteases, MT1-MMP, MMP2 and MMP9. Additionally, we assessed the effect of TNF-α on alterations in the concentrations of all these proteases in detergent resistant membranes (DRMs) with emphasis on the role of the MAPK/ERK signaling pathway in this process.

## Materials and methods

### Cell culture

MDA-MB-231 and MCF7 breast cancer-derived cells were cultured in DMEM (Lonza, Verviers, France), supplemented with 10 % fetal bovine serum (FBS) (Lonza), 1 % glutamine, 100 U/ml penicillin and 100 mg/ml streptomycin at 37 °C in a 5 % CO_2_ humidified incubator. The cells were seeded in 12-well plates or 10 cm Petri dishes and maintained until they reached 70–90 % confluency. Next, the cells were starved overnight in serum-free DMEM and subsequently stimulated with 10 ng/ml TNF-α for 24 h. No TNF-α was added to the control. Conditioned medium (CM) was collected for substrate zymography. The cells were lysed for both total RNA and protein extractions (see below).

### Reagents and antibodies

TNF-α was obtained from ProSpec-Tany TechnoGene Ltd. (cyt-223-b) and U0126 was purchased from Santa Cruz (CAS 109511–58-2). The antibodies used were: rabbit anti-phospho-ERK1/2 (T202/Y204, Cell Signaling), mouse anti-ERK1/2 (L34F12, Cell Signaling), rabbit anti-MMP-9 (AB19016, Merck Millipore), rabbit anti-MMP2 (VARP20016_T100, Aviva), rabbit anti-MMP14 (PA5–13,183, Thermo Scientific), rabbit anti-FAP-α (GTX102732, GeneTex), goat anti-DPPIV/CD26 (AF1180, R&D Systems), rabbit-anti-Cortactin (H-191) (sc-11,408, Santa Cruz Biotechnology, Inc.), goat anti-actin (I-19) (sc-1616, Santa Cruz Biotechnology, Inc.) and mouse anti-FL-2 (B6) (sc-28,320, Santa Cruz Biotechnology, Inc.). The cholera toxin subunit B-HRP conjugate was obtained from Molecular Probes (C-34,780). Millicell Hanging Cell Culture Inserts (8.0 μm) were purchased from Merck Millipore (PIEP15R48) and blotting membranes were purchased from Bio-Rad (162–0094). Immuno-detection was performed using HRP-conjugated donkey anti-rabbit (sc-2313), donkey anti-goat (sc-2020) and goat anti-mouse (sc-2005) antibodies purchased from Santa Cruz Biotechnology, Inc. and Pierce™ECL Western Blotting Substrate (32,106, Thermo Scientific).

### Semi-quantitative reverse transcription polymerase chain reaction (RT-PCR)

Total RNA isolation was performed using a RNeasy kit (Qiagen, USA). All the RT-PCR reactions were performed using a one-step RT-PCR kit (Clontech Laboratories, Inc., A Takara Bio Company), according to the manual provided by the manufacturer. For RT-PCR, 1 μl of the total RNA extracted from each sample was added to a 20 μl final reaction volume of RT-PCR mix containing 0.25 pmol of each specific primer. Reverse transcription (RT) was performed for 1 h at 50 °C and 5 min at 94 °C. To amplify the FAP-α transcripts, we used primer pair 5′-GCT GGA GCT AAG AAT CCC GTT GTT CG-3′ (sense) and 5′-TGC TTG GAG GAT AGC TTC CAA TGC T-3′ (antisense). The FAP-α amplification product length was 544 bp. The PCR conditions were 30 s at 94 °C, 45 s at 60 °C and 1 min at 68 °C (30 cycles). To detect genomic DNA contamination, RT-free PCR controls were included. To amplify the CD26 (473 bp) and MMP-14 (395 bp) transcripts we used the primer pairs 5′- GGA AGA TGG AAC TGC TTA GTG GCA CG -3′ (sense) and 5′- TCT CAG CCC TTT ATC ATT CAC GCT GC -3′ (antisense), and 5′-TCA TGA TCT TCT TTG CCG AGG GCT-3′ (sense) and 5′-TTT ATC AGG AAC AGA AGG CCG GGA-3′ (antisense), respectively. The PCR products were separated and visualized in 1 % agarose gels containing ethidium bromide, digitized, and assessed for densitometry using ImageJ software. For semi-quantitative analyses the transcripts were related to co-amplified actin, a housekeeping control transcript. The actin primers used were 5′-TAC AAT GAG CTG CGT GTG GCT CCC CCC G-3′ (sense) and 5′-AAT GGT GAT GAC CTG GCC GTC AGG C-3′ (antisense) applied under the same amplification conditions as described above, yielding a 479 bp product. The pixel density of each individual PCR product was calculated relative to the pixel densities of the actin transcripts, the values of which were set at 1.

### Isolation of DRM and DSM membrane fractions

For membrane micro-domain fractionation, 15 x 10^6^ cells were lysed for 30 min on ice in 1 % Triton X-100 in TNE buffer (150 mM NaCl, 1 mM EDTA, 25 mM Tris, pH 7.4) containing a protease inhibitor cocktail and ultra-centrifuged on a discontinuous 5 %/30 %/40 % sucrose gradient. To this end, the cell lysates were mixed 1:1 with 85 % sucrose in TNE buffer containing 1 % Triton X-100. This mixture (1 ml) was placed in the bottom of a 4.5 ml centrifuge tube. Next, 2.7 ml of 30 % sucrose was poured on the top of the diluted lysate and subsequently overlaid with 0.8 ml of 5 % sucrose in TNE buffer. The samples were centrifuged in a Ti60 rotor (Beckman) at 35,000 RPM at 4 °C for 16 h. Eight 550 μl sucrose gradient fractions were collected from the top to the bottom of the tube and assessed for GM1 ganglioside and total protein content by dot blotting using a peroxidase-linked cholera toxin B subunit (CTX-HRP) and Ponceau S, and for flotilin-2 by immunoblotting. Low-density fractions containing detergent resistant membranes (DRM) are known to be enriched in GM1 and flotilin-2 in contrast to high-density fractions, which contain detergent soluble membranes (DSM) and have a notably lower concentration of both of these factors.

### SDS-PAGE and Western blotting

For SDS-PAGE, proteins in each fraction (see above) were precipitated with 10 % trichloroacetic acid (TCA), washed twice with cold acetone and suspended in 50 μl (4×) Laemmli sample buffer. All the samples were heated at 95 °C for 10 min and electrophoresed (10 μl/slot) through 10 % SDS polyacrylamide gels. After electrophoresis, the samples were transferred overnight at 95 mA to nitrocellulose membranes. These membranes were subsequently blocked for 1 h with 5 % non-fat milk in PBS (pH 7.4) at room temperature and then incubated overnight at 4 °C with primary antibodies. Semi-quantitative digital image analyses were performed using ImageJ software to detect the integrated intensities of particular bands. The results are expressed as ratios of integrated density values of corresponding protein bands from treated to untreated control cells.

### Gelatin zymography

For the analysis of gelatinolytic activity, sucrose gradient fractions as well as conditioned media were mixed 1:0.5 *v*/v with sample buffer (62.5 mMTris-HCl, pH 6.8, with 10 % glycerol, 2 % SDS and 0.05 % bromophenol blue). A total of 30 μl of each of the non-reduced samples was loaded in 10 % SDS-polyacrylamide gels copolymerized with gelatin (2 mg/ml). After semi-native electrophoresis, the enzymes were renatured by washing out the SDS twice in 50 mM Tris-HCl (pH 7.5) with 2.5 % Triton X-100 for 30 min at room temperature. Next, the gels were incubated for 20 h at 37 °C in 50 mM Tris-HCl (pH 7.5) containing 150 mM NaCl, 10 mM CaCl2, 1 μM ZnCl2 and 0.05 % Brij-35, stained with 0.12 % Coomassie blue and de-stained with a solution containing 5 % acetic acid and 10 % ethanol in water.

### Transwell migration assay

2.5 x 10^4^ MDA-MB-231 and MCF7 cells suspended in 1 ml serum-free medium were loaded onto a upper 8 μm pore size chamber inserted in a 12-well cell culture plate. The lower chamber was filled with DMEM supplemented with 1 % FBS as a chemoattractant*.* The cells were allowed to adhere before being treated with 10 ng/ml TNF-α and then incubated for 24 h at 37 °C in 5 % CO_2_. After this incubation, the inserts were removed and the remaining non-migrating cells on the upper surface of the membrane were removed with a cotton swab. The cells that migrated to the lower surface of the membrane were fixed with 5 % paraformaldehyde (PFA) in PBS for 20 min at room temperature, washed with PBS and then treated with 0.5 % Triton X-100 for 5 min. Next, the cells were stained with DAPI solution (Mounting Medium with DAPI, Sigma-Aldrich) and examined using a Zeiss LSM 510meta microscope at 20× magnification (Plan Apochromat 20×/0,8). Pictures of five randomly chosen fields were taken and migrating cells were counted using ImageJ software. The average number of migrating cells for each condition was calculated and differences in the numbers of cells that migrated through the membrane were calculated.

### Wound healing assay

MDA-MB-231 and MCF7 cells were seeded in Petri dishes and cultured to reach 90 % confluency in complete medium. Next, the cells were starved overnight and a sterile 200 μl pipet tip was used to scrape four wounds through the cell monolayer. The cells were gently rinsed before being treated with 10 ng/ml TNF-α. Pictures were taken immediately before TNF-α application and after 24 h using phase contrast optics. Images were acquired with a 10×/1.25 numerical aperture objective on an inverted microscope (Zeiss) equipped with a digital camera using Image-Pro Plus version 5.1.2.59. Wound areas were quantified using imageJ software version 1.43u. The results are presented as wound closure rates.

## Results

### TNF-α affects MMP9 protein level and secretion in breast cancer-derived cells

**Fig. 1:**
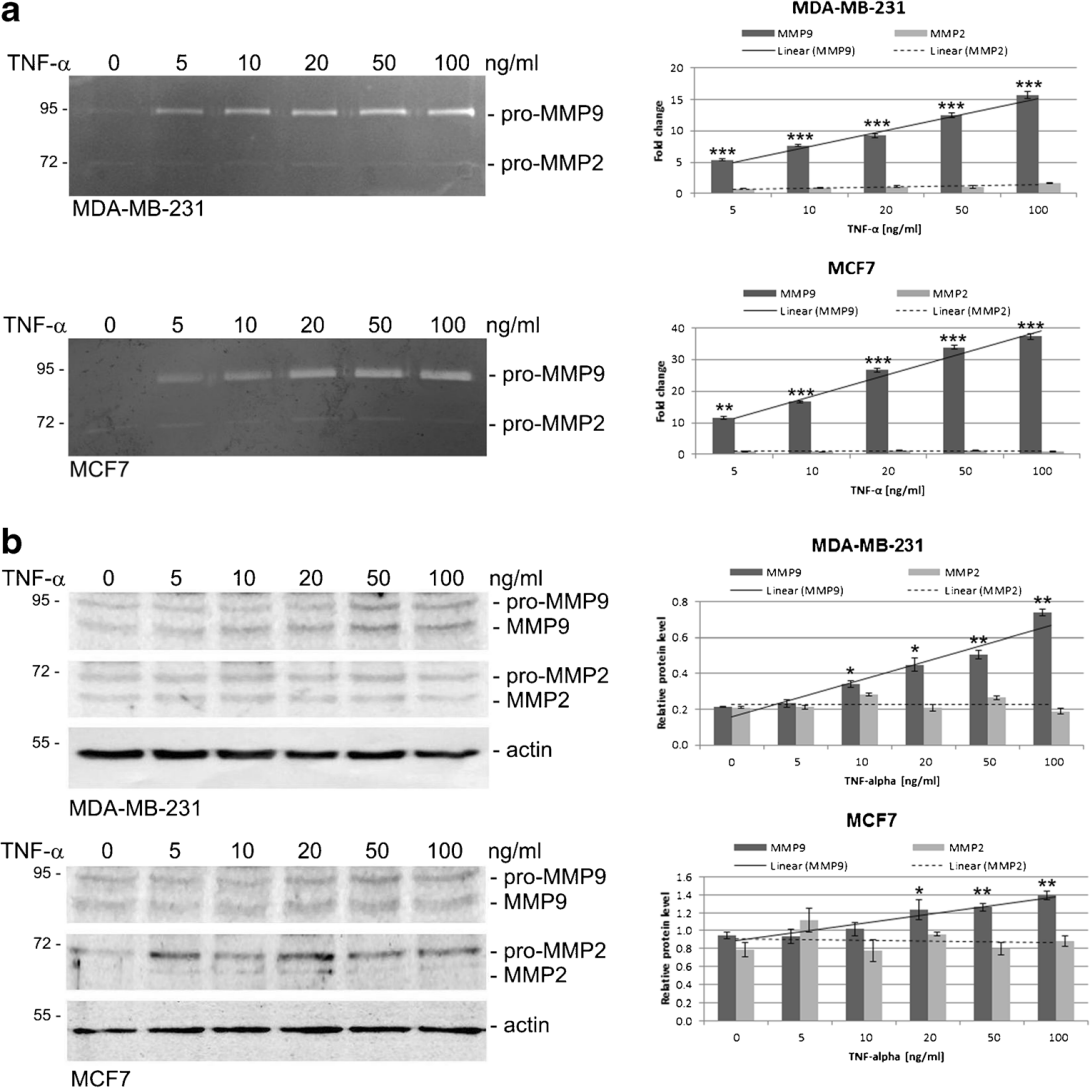
TNF-α induces increases in MMP9, but not MMP2, synthesis and activity in breast cancer cells. **a** MDA-MB-231 and MCF7 cells were cultured in the presence of TNF-α at the indicated concentrations for 24 h. Conditioned media were collected and subjected to analysis of secreted gelatinases by gelatin zymography. Electrophoretic bands were quantified by densitometry and normalized to the untreated controls. Bar graphs present the relative fold change in MMP9 and MMP2 activity in relation to TNF-α concentration. **b** Total cell lysates were prepared from untreated or TNF-α treated cells for 24 h at the concentration indicated and immunoblotted using anti-MMP9 and anti-MMP2 antibodies. Protein bands were quantified by densitometry and normalized to the corresponding actin (housekeeping protein) band. Bar graphs present the relative fold change in protein level for MMP9 and MMP2. All data are presented as mean ± SEM from at least three independent experiments (*n* ≥ 3). *P < 0.05, **p < 0.005, ***p < 0.0001 compared to control (untreated) cells

To examine the effect of TNF-α on gelatinase production and secretion, we treated MDA-MB-231 and MCF7 cells with TNF-α at the indicated concentrations for 24 h in serum-free media. A dose-dependent increase in the MMP9 concentration in conditioned media and in cell lysates was observed. A strong cellular response was seen in the TNF-α concentration range of 10–100 ng/ml (Fig. 1a). In case of MMP2, non-significant changes in protein level after TNF-α treatment were observed in cell lysates by Western blotting as well as in conditioned media by gelatin zymography (Fig. 1a-b).

### TNF-α affects breast cancer-derived cell migration

**Fig. 2:**
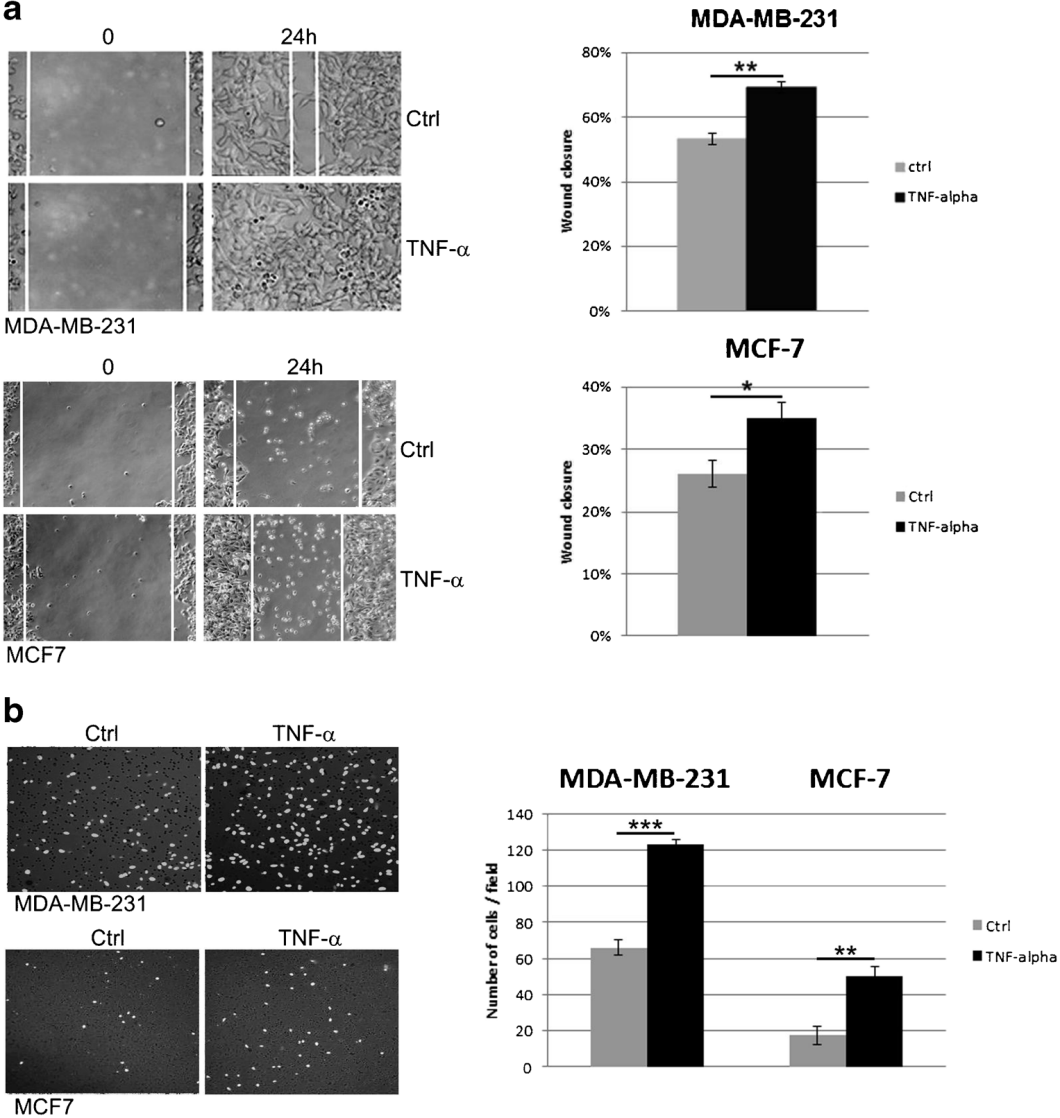
Effect of TNF-α on cell migration. **a** Cell migration evaluated by scratch wound healing assay. Cells were treated with TNF-α or left untreated (control). Images were taken at 0 and 24 h after wound formation, and the wound surface areas were measured using Image J software and normalized to the initial state. Representative phase-contrast images of MDA-MB-231 and MCF-7 cells migrating into the wounded area are shown (left). Bar graphs present relative changes in wound closure for cells treated and untreated with TNF-α. **b** Cell migration evaluated by transwell migration assay. Representative images of migrating cells on the bottom of Millicell’s membranes (pore size 8 μm) in the presence (right panel) and in the absence of TNF-α after 24-h incubation are shown. Bar graphs represent number of transmigrating cells in TNF-α treated and untreated cell cultures. All data are presented as mean ± SEM from three independent experiments (*n* = 3). *P < 0.05, **p < 0.005 ***p < 0.0001 compared to control (untreated) cells

Using a wound healing assay and a Transwell cell migration assay we found that TNF-α enhanced the migration of both MDA-MB-231 and MCF-7 cells. As shown in Fig. 2a the efficiency of the wound closure was significantly lower in the untreated control cells compared to the TNF-α stimulated cells. We found that TNF-α at a concentration of 10 ng/ml enhanced wound closure by ∼6 % and ~9 % at 24 h compared to the control (untreated) MDA-MB-231 and MCF-7 cells, respectively. This enhanced migration after TNF-α treatment was confirmed by an independent Transwell assay in which the number of cells that migrated across 8 μm diameter pores over 24 h was counted. The data of the Transwell assay are depicted in Fig. 2b.

### TNF-α affects CD26, FAP-α and MT1-MMP expression

**Fig. 3:**
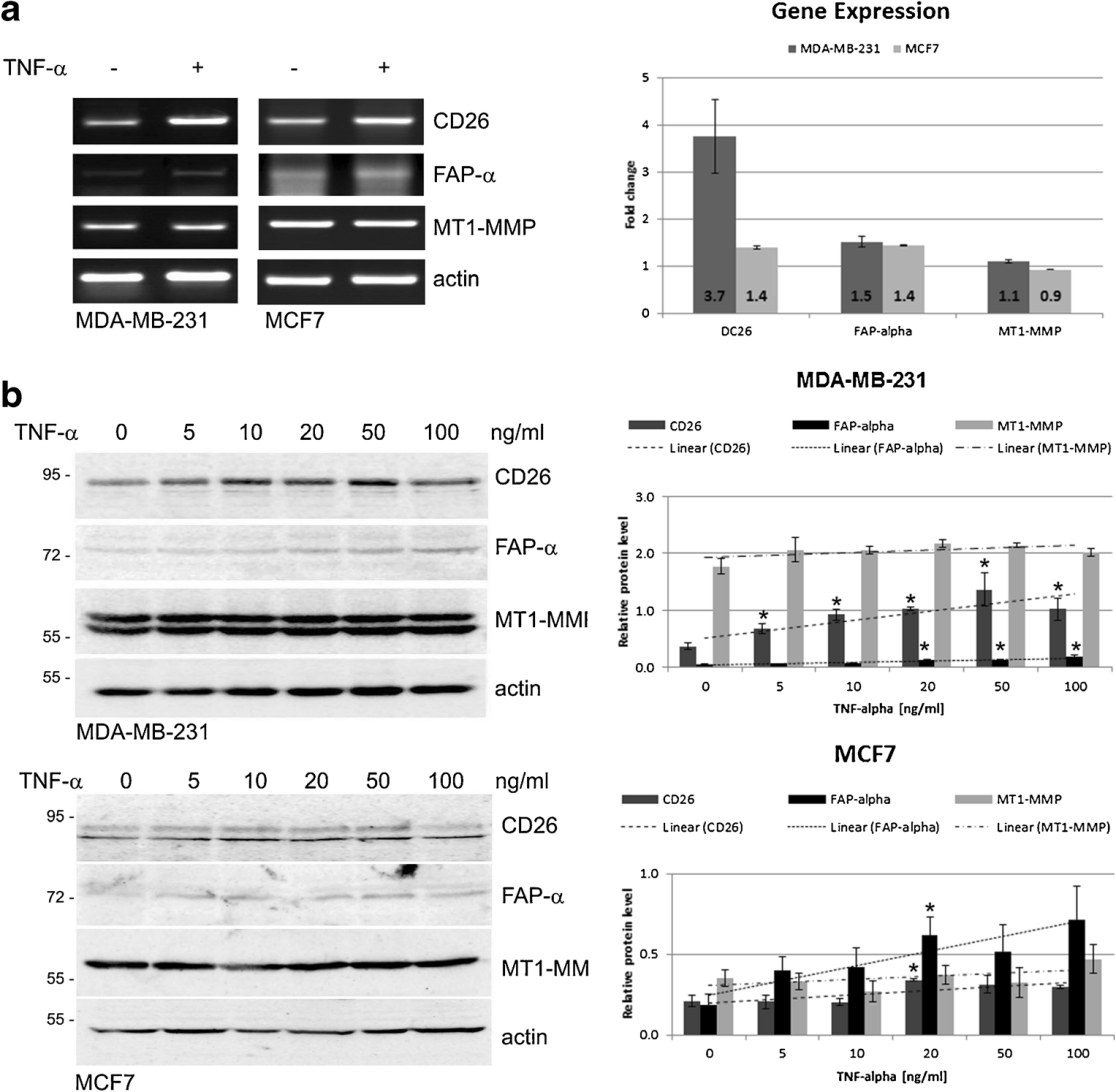
Effect of TNF-α on CD26, FAP-α and MT1-MMP protein and mRNA expression levels. **a** MDA-MB-231 and MCF7 cells were stimulated with TNF-α (10 ng/ml) or left unstimulated for 24 h after which total RNA was isolated. Levels of CD26, FAP-α and MT1-MMP transcripts were analyzed by RT-PCR using gene-specific primers followed by agarose gel electrophoresis. Electrophoretic bands were quantified by densitometry and normalized to the housekeeping gene included (actin). Bar graphs present relative fold changes in gene expression for CD26, FAP-α, and MT1-MMP in both cell lines. **b** Total cell lysates were prepared from untreated or TNF-α treated cells for 24 h at the concentration indicated and immunoblotted using anti-CD26, anti-FAP-α and anti-MT1-MMP antibodies. All protein bands were quantified by densitometry and normalized to the corresponding actin (housekeeping protein) band. Bar graphs represent the fold changes in protein levels for CD26, FAP-α and MT1-MMP in relation to TNF-α concentration. All data are presented as mean ± SEM from at least three independent experiments (*n* ≥ 3). *P < 0.05 compared to control (untreated) cells

Initially, we set out to compare the CD26, FAP-α and MT1-MMP mRNA expression levels in cells treated with 10 ng/ml TNF-α for 24 h with those in untreated cells by RT-PCR. Although the mRNA transcript levels of all these genes were found to increase in cells stimulated with TNF-α compared to unstimulated control cells (Fig. 3a), we found that TNF-α had only a slight impact on the expression of FAP-α and MT1*-*MMP. Similar effects were observed in both the MDA-MB-231 and the MCF-7 cells. These findings were confirmed by examining the respective protein levels by Western blotting. By analyzing the pixel intensities of the protein bands normalized to actin, we found that all three proteins were upregulated in a dose-dependent manner (Fig. 3b). Substantial changes were, however, only observed for CD26. Statistically significant increases in FAP-α protein levels were also observed, but only in cells exposed to the higher concentrations of TNF-α (20–100 ng/ml).

### TNF-α induces localization of membrane-associated proteases into lipid rafts

**Fig. 4:**
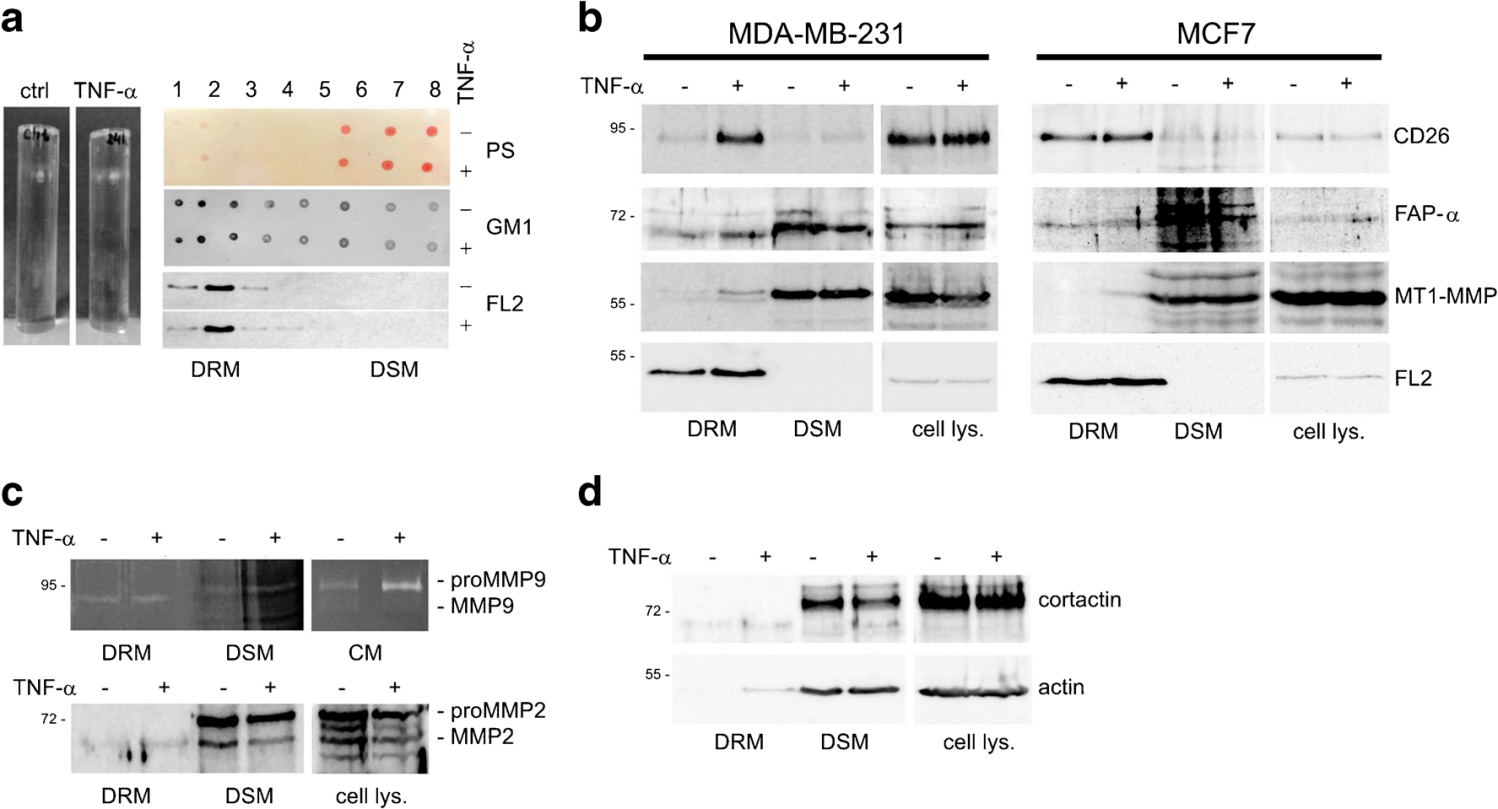
TNF-α-induced accumulation of membrane-associated proteases in lipid rafts. **a** Discontinuous sucrose density gradient ultracentrifugation was used to isolate detergent resistant membranes (DRM) and detergent soluble membranes (DSM) from TNF-α treated or untreated MDA-MB-231 and MCF7 cells at 10 ng/ml for 24 h. Fractions (1–8) were collected from the top of the gradient and the distribution of the lipid raft markers, glycosphingolipid (GM1) and Flotillin 2 (FL2), was determined by dot blotting and Western blotting using HRP-conjugated cholera toxin (middle panel) and a specific antibody directed against Flotillin 2 (FL2) (bottom panel). Total proteins in sucrose gradient fractions were visualized by Ponceau*-*S (PS) staining (top panel). **b** Visualization of membrane-associated proteases in cell lysates as well as in DRM and DSM fractions derived from TNF-α treated cells and untreated control cells by immunoblotting. TNF-α stimulation of MDA-MB-231 and MCF7 cells induces increases in CD26 (fold change: 8.01 and 1.26), FAP-α (fold change: 1.68 and 2.03) and MT1-MMP (fold change: 3.84 and 25.47) concentrations, respectively in the DRM fraction.**c** Distribution of MMP9 and MMP2 to DRM and DSM fractions in the presence or absence of TNF-α was analyzed by gelatin zymography and Western blotting. Both gelatinases are slightly increased (fold change: 1.23 and 1.37 for MMP9 and MMP2, respectively) in the DRM fraction after TNF-α induction. **d** Changes in the concentration of the invadopodia-associated proteins actin and cortactin in DRM fractions in cells treated with TNF-α were observed by immunoblotting. The fold changes in protein levels for cortactin and actin are 2.02 and 24.87, respectively. No differences in the total levels of these proteins under TNF-α stimulation (cell lysate panel) were observed

Sucrose gradient fractions were analyzed by Western blotting for flotillin-2 content and by dot blotting, using a peroxidase-linked cholera toxin B subunit, for GM1 content to identify DRM fractions that contain lipid raft material. The highest concentrations of flotillin-2 and GM1 were found in fraction 2. Therefore, we here refer to fraction 2 as a DRM fraction and to fraction 7, which is low in DRM markers, as a DSM fraction (Fig. 4a).

To determine whether membrane-associated proteases are localized into lipid rafts under TNF-α stimulation we determined the levels of FAP-α, CD26, MT1-MMP in DRM fractions, isolated from MDA-MB-231 and MCF7 cells untreated and treated with 10 ng/ml TNF-α for 24 h, by Western blotting. In Fig. 4b the distribution is shown of three transmembrane proteases, FAP-α, CD26 and MT1-MMP, in total cell lysates and in the low-density (DRM) as well as high-density (DSM) fractions derived from cells treated with TNF-α or untreated control cells. We found increased levels of all these proteins in the DRM fractions of treated cells as compared to those of untreated control cells (both cell types). In the DRM fractions of the TNF-α treated cells increased levels of MMP9 and MMP2 were also observed by gelatin zymography and Western blotting. As shown in Fig. 4c, only active forms of gelatinases were localized in the DRM.

To confirm that the TNF-α-induced changes observed in the localization of proteases in the lipid rafts are related to cell migration, we assessed the levels of actin and cortactin in the sucrose gradient fractions from treated and untreated (control) cells. These two proteins are known to be tightly associated with the formation of invadopodia, membrane protrusions that are characteristic for highly invasive cancer cells. Indeed, we found that also the concentrations of actin and cortactin increased in the DRM fractions of MDA-MB-231 and MCF7 cells treated with TNF-α (Fig. 4d).

### U-0126 affects TNF-α-induced localization of proteases in lipid rafts through the MAPK/ERK pathway

**Fig. 5:**
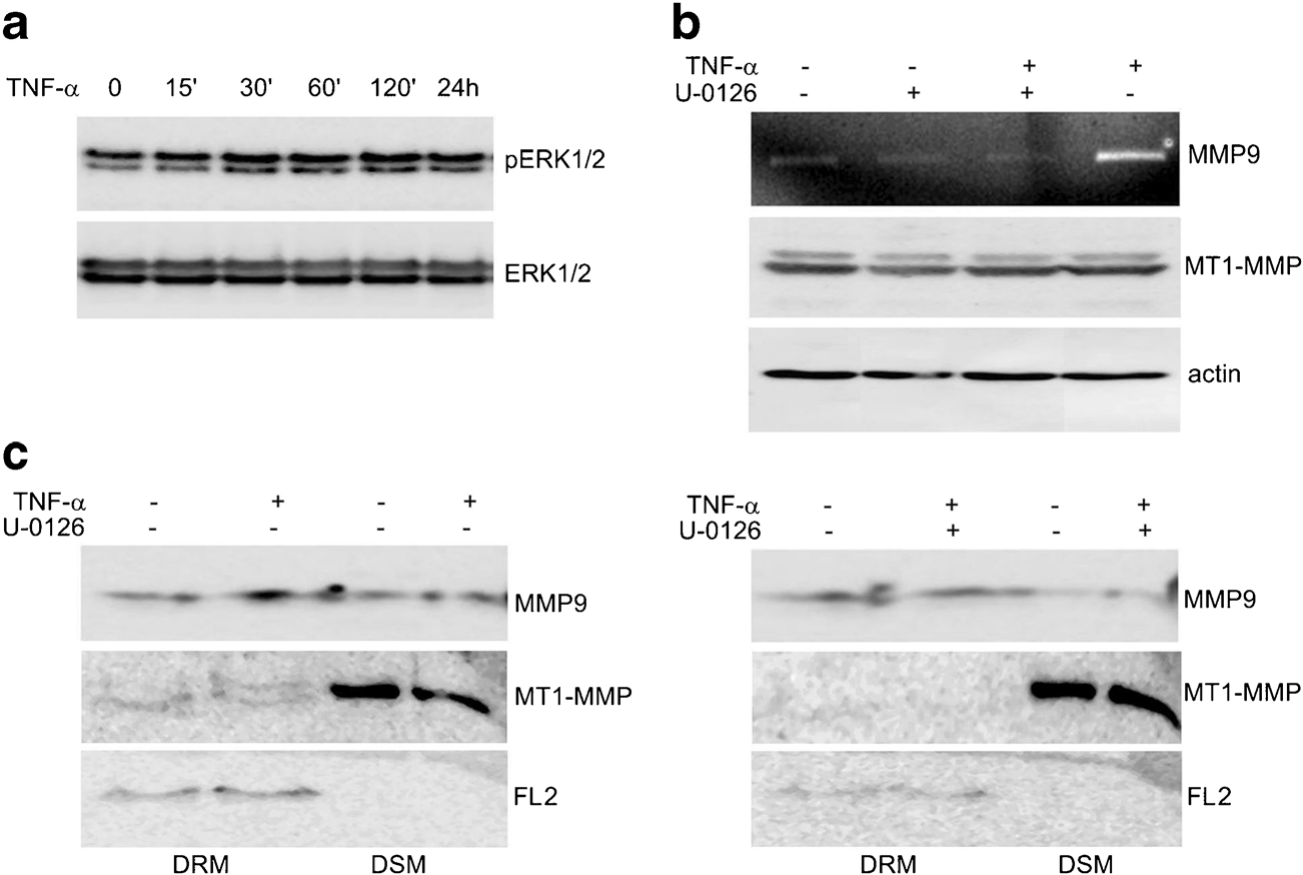
TNF-α stimulation of MDA-MB-231 cells increases accumulation of proteases in lipid rafts through the MAPK/ERK pathway*.* **a** MAPK/ERK activation was assayed by immunoblotting for phospho-ERK1/2 (pERK1/2 panel) in cells treated with TNF-α (10 ng/ml) for the indicated times. Blots were stripped and re-probed for total ERK1/2 (ERK1/2 panel). **b** MDA-MB-231 cells were either left untreated or pretreated with the MEK/ERK inhibitor, U-0126 (10 μM), for 1 h. Next, the cells were stimulated with TNF-α (10 ng/ml) for 24 h. Levels of secreted MMP9 in the culture media were assayed by gelatin zymography (top panel) and the levels of MT1-MMP in cell lysates were analyzed by Western blotting (middle panel). Actin was used as a loading control (bottom panel). **c** The concentration of MMP9 and MT-MMP in DRM and DSM fractions isolated from cells that were either pretreated or not with 10 μM U-0126 and, subsequently, stimulated with TNF-α (10 ng/ml) for 24 h was determined by immunoblotting. Flotillin 2 (FL2) served as a marker of DRM

Through Western blot analyses of MDA-MB-231 cell lysates we observed a substantial increase in ERK1/2 phosphorylation after TNF-α treatment (Fig. 5a). U-0126 is known to selectively block the activity of the MAP kinase MEK, which is an activator of the ERK1/2 kinases. After pretreatment of MDA-MB-231 cells with U-0126 (10 μM) we observed an abrogation TNFα- induced MMP9 secretion in conditioned media (Fig. 5b). Western blot analyses of MMP9 in DRM fractions from cells pretreated with U-0126 and treated with TNF-α revealed that inhibition of the MAPK/ERK pathway blocks the TNF-α-induced increases in protease concentrations in the DRM fractions (Fig. 5c). Similar results were obtained for MT1-MMP, the protease of which the total levels were not significantly changed after TNF-α treatment.

## Discussion

Tumor necrosis factor α (TNF-α) is a key cytokine that is involved in the generation of inflammatory responses. Although TNF-α was initially found to induce cell death in some cancer types, recent studies have shown that it may also exert tumor-promoting activities such as the induction of transformation, proliferation, angiogenesis, invasion and metastasis [6]. A high level of TNF-α is a characteristic of many malignant cancers, including breast cancers, and has frequently been associated with an aggressive behavior and a poor prognosis [3]. TNF-α is primarily produced by macrophages, but it can also be produced by a variety of other stromal cells, as well as cancer cells themselves. Thus, it may act in both a paracrine and an autocrine manner [24]. TNF-α is known to play a particular role in enhancing tumor cell migration and invasion, but the underlying mechanisms are still elusive [4, 24].

Here, different in vitro assays were employed to assess the effect of TNF-α on breast cancer cell migration. We found that TNF-α strongly promoted the migration of both hormone-dependent and highly invasive, triple-negative, breast cancer cells using a Transwell assay and to accelerate wound closure using a scratch assay. These results are in keeping with studies reporting the pro-migratory activity of TNF-α on melanoma cells, bladder cancer cells, cervical cancer cells and leukemia cells [4, 25–27]. In our current study, TNF-α-induced cell migration was accompanied by an increased secretion of MMP9, but not MMP2, into the culture media. MMP2 is known to be expressed at a constant level by various cell types, while the synthesis and secretion of MMP9 is known to be tightly regulated by different biological factors [8, 27]. TNF-α is considered to be one of the strongest physiological inducers of MMP9 expression, the enzyme which appears necessary for sustained migration of various cell types, including cancer cells [28–30]. MMP9 was found by others to be implicated in promoting breast cancer cell migration and invasion through interactions with integrins and non-integrin type receptors such as CD44 [31]. Using MMP9 knockout mice, it was found that MMP9 is essential for the migration of smooth muscle and immune cells, and that disruption of MMP9-cell surface interactions can cause a dramatic decrease in cell movement and tumor invasiveness [32, 33].

Here, we found that also one of dipeptidyl peptidases, CD26, becomes significantly upregulated in response to TNF-α. In parallel, we observed a markedly increased uptake of all studied proteases into lipid rafts after TNF-α treatment, regardless whether TNF-α-induced changes in mRNA or protein levels were relatively high or only minor, as in the case of MT1-MMP.

Recently, it was postulated that lipid rafts may play a critical role in cancer cell adhesion and migration [18, 34]. Lipid rafts, which are cholesterol- and sphingolipid-rich membranes resistant to non-ionic detergents, appear to be directly associated with various cellular adhesion structures such as focal contacts and invadopodia and, thereby, essential for cell-ECM interactions and matrix degradation. Membrane rafts have been described in numerous malignant cancer models, including breast cancer models, as being highly ordered membranous domains determining the activation of cell survival signaling pathways. These domains were also found to be the basis for resistance to targeted cancer therapy and, accordingly, disruption of the membrane raft structure has been found to decrease this resistance in cancer cells [18]. Lipid rafts have been found to be concentrated at the leading edge of invadopodia in different types of breast cancer cells, and direct manipulation of cholesterol levels with methyl-β cyclodextrin (MβCD) or statins was found to impair focal adhesion disassembly as well as invadopodia formation and their functions [18, 21, 34, 35]. Invadopodia are specialized F-actin and cortactin-rich membrane protrusions formed at the ventral surface of highly aggressive cancer cells, including MDA-MB-231, and display local proteolytic activity towards extracellular matrix constituents. Their high lytic activity seems to be due to the accumulation of proteolytic enzymes at the sites of active actin polymerization [34–36]. A number of key invadopodial components, including proteolytic enzymes such as MT1-MMP and CD26, have been found to reside in lipid rafts [18, 21, 37–39].

Here, we found that exogenous TNF-α enhances the accumulation of both MT1-MMP and CD26 and, additionally, FAP-α in lipid rafts in different breast cancer-derived cells. Moreover, we found that lipid rafts of TNF-α-treated cells were also enriched in two gelatinases, MMP9 and MMP2. MMP2 and MMP9 are secretory proteins, but small amounts of the enzymes have consistently been found on the surface of various cells, including breast cancer cells [21, 40]. In contrast to the DSM fractions in which both pro-enzymes and active forms of MMP9 and MMP2 were identified, we only found active enzymes in the DRM fractions, regardless whether TNF-α was added to the culture media or not. This finding suggests a contribution of lipid rafts in the activation of membrane-associated gelatinases. Since cancer cell migration and invasiveness are thought to correlate with the presence of actin- and cortactin-based protrusions, we next set out to assess changes in the accumulation of both these proteins in lipid rafts of breast cancer-derived cells stimulated with TNF-α to establish a potential contribution of invadopodia in TNF-α-induced cell migration. Recently, TNF-α was shown to promote invadopodia formation in MDA-MB-231 cells [41]. Here, we found that the concentrations of actin and cortactin were altered in lipid rafts of TNF-α stimulated cells. However, in the case of cortactin, only a shortened form of the protein, corresponding to cortactin splice variant 1 (SV1), could be detected in the DRM fraction.

A number of different growth factors and cytokines, including TNF-α, have been found to regulate MMP9 expression via the extracellular signal-regulated kinase (ERK) [26, 42, 43]. It was previously found that granulocyte colony stimulating factor (G-CSF) is able to increase MT1-MMP gene expression and protein synthesis in hematopoietic cells and to promote partitioning of MT1-MMP into lipid rafts through a mechanism that is regulated by the phosphatidylinositol 3-kinase (PI3K) signaling pathway [39]. Here, we showed that the TNF-α-enhanced location of proteases in lipid rafts is dependent on ERK signaling pathway activation. We also showed that the application of U-0126, a specific MEK/ERK inhibitor, led to an effective block of the TNF-α-induced accumulation of MMP9 and MT1-MMP in lipid rafts. Since we found that TNF-α has only a minor effect on MT1-MMP mRNA and protein expression levels, its role in the enhancement of protease uptake into lipid rafts seems to be independent of its role as an inducer of protease gene expression.

Taken together, we conclude that TNF-α-induced enhancement of breast cancer cell migration is accompanied by increased concentrations of proteases that are directly (MT1-MMP, CD26 and FAP-α) and indirectly (MMP9 and MMP2) associated with the cellular membrane in lipid rafts through activation of the MAPK/ERK signaling pathway. Despite the notion that the localization and concentration of protease activity in the lipid platforms may be crucial for various biological processes, including cell migration and invasion, its exact underlying molecular mechanisms have so far not been fully elucidated and understood. Further studies may comprehend this complex process and reveal suitable therapeutic targets.
